# Case Report: Cytokeratin-positive interstitial reticulum cell tumor with HLA loss of heterozygosity

**DOI:** 10.3389/fimmu.2026.1844173

**Published:** 2026-06-02

**Authors:** Wen Liu, Yan Zhang, Yuqing Wei, Yinan Hu, Xinmiao Zhang, Jingli Xue, Peifeng Li

**Affiliations:** 1 Department of Pathology, The 960th Hospital of the People’s Liberation Army (PLA) Joint Logistic Support Force, Jinan, China; 2 Department of General Surgery, The 960th Hospital of the People’s Liberation Army (PLA) Joint Logistic Support Force, Jinan, China

**Keywords:** case report, cytokeratin-positive interstitial reticulum cell tumor, fibroblastic reticular cell tumor, immune evasion, loss of heterozygosity of human leukocyte antigen, stroma-derived neoplasms of lymphoid tissues

## Abstract

**Introduction:**

Cytokeratin−positive interstitial reticulum cell (CIRC) tumor, a subtype of fibroblastic reticular cell tumor (FRCT), is an extremely rare primary neoplasm of lymph nodes and soft tissue, with limited understanding of its clinicopathological and molecular features. This case is the first identification of human leukocyte antigen loss of heterozygosity (HLA LOH) in CIRC tumor, which provides novel insights into immune evasion mechanisms and potential therapeutic implications.

**Case presentation:**

A 67−year−old female presented with a local recurrence seven years after initial resection of a CIRC tumor on her right shoulder. Physical examination revealed a firm, poorly mobile subcutaneous mass (12cm×8cm). Imaging confirmed a right parascapular mass with bone destruction. Histologically, the recurrent tumor consisted of spindle and epithelioid cells arranged in storiform and sheet−like patterns, with extensive necrosis and a mitotic count of 3 per 10 high−power fields. Immunohistochemically, tumor cells diffusely expressed cytokeratins, vimentin, CD68, CD163, and EMA, with focal expression of SMA, S−100, calponin, and CD3, but were negative for CD21, CD35, CD1a, ALK, and HHV8. The Ki−67 index was 25%. Whole−exome sequencing identified 30 single−nucleotide variants and 7 indels (variant allele frequencies 2.17%–12.6%), copy number gains on chromosomes 7, 11, and 14, and microsatellite stability. Notably, two HLA LOH events affecting HLA−B39:01:01:01 and HLA−C07:02:01:01 were detected. No disease progression was observed during follow−up.

**Conclusion:**

This is the first report of HLA LOH in FRCT/CIRC tumor. The key take−away lesson is that HLA LOH represents a potential immune evasion mechanism, which may render single−agent immune checkpoint inhibitors ineffective and thus guide alternative immunotherapeutic strategies. Chromosomal instability appears to be a prominent genomic feature of FRCT. These findings expand the molecular landscape of this rare tumor and underscore the value of comprehensive genomic profiling in guiding individualized treatment.

## Introduction

Fibroblastic reticular cell tumor (FRCT) is an extremely rare neoplasm originating from stromal cells of lymphoid tissues (1, 2). The 5th edition of the World Health Organization (WHO) Classification of Haematolymphoid Tumors has reclassified FRCT-together with follicular dendritic cell sarcoma-from the category of “histiocytic and dendritic cell neoplasms” into a new category designated “stroma-derived neoplasms of lymphoid tissues” (1–3). This revision reflects the growing recognition that fibroblastic reticular cells are not of hematopoietic origin but rather derive from mesenchymal cells. Based on cytokeratin expression status, fibroblastic reticular cell tumors (FRCTs) can be further subclassified into cytokeratin-negative and cytokeratin-positive subtypes, with the latter also referred to as cytokeratin-positive interstitial reticulum cell (CIRC) tumor (4–6).The prognosis and clinical outcomes of FRCT exhibit significant heterogeneity, with its biological behavior ranging from “in situ” or indolent to highly aggressive behavior (5, 7–12). Currently, the understanding of FRCT remains limited, and its molecular characteristics have not yet been fully elucidated (4, 13).

In this study, we performed a retrospective clinicopathological analysis of a CIRC tumor case. Whole exome sequencing (WES) and RNA sequencing were performed to investigate its genetic characteristics. The case report enhances understanding of the molecular characteristics of FRCT, and identifies human leukocyte antigen loss of heterozygosity (HLA LOH) as a potential biomarker to inform individualized treatment decisions.

## Case presentation

**Figure 1:**
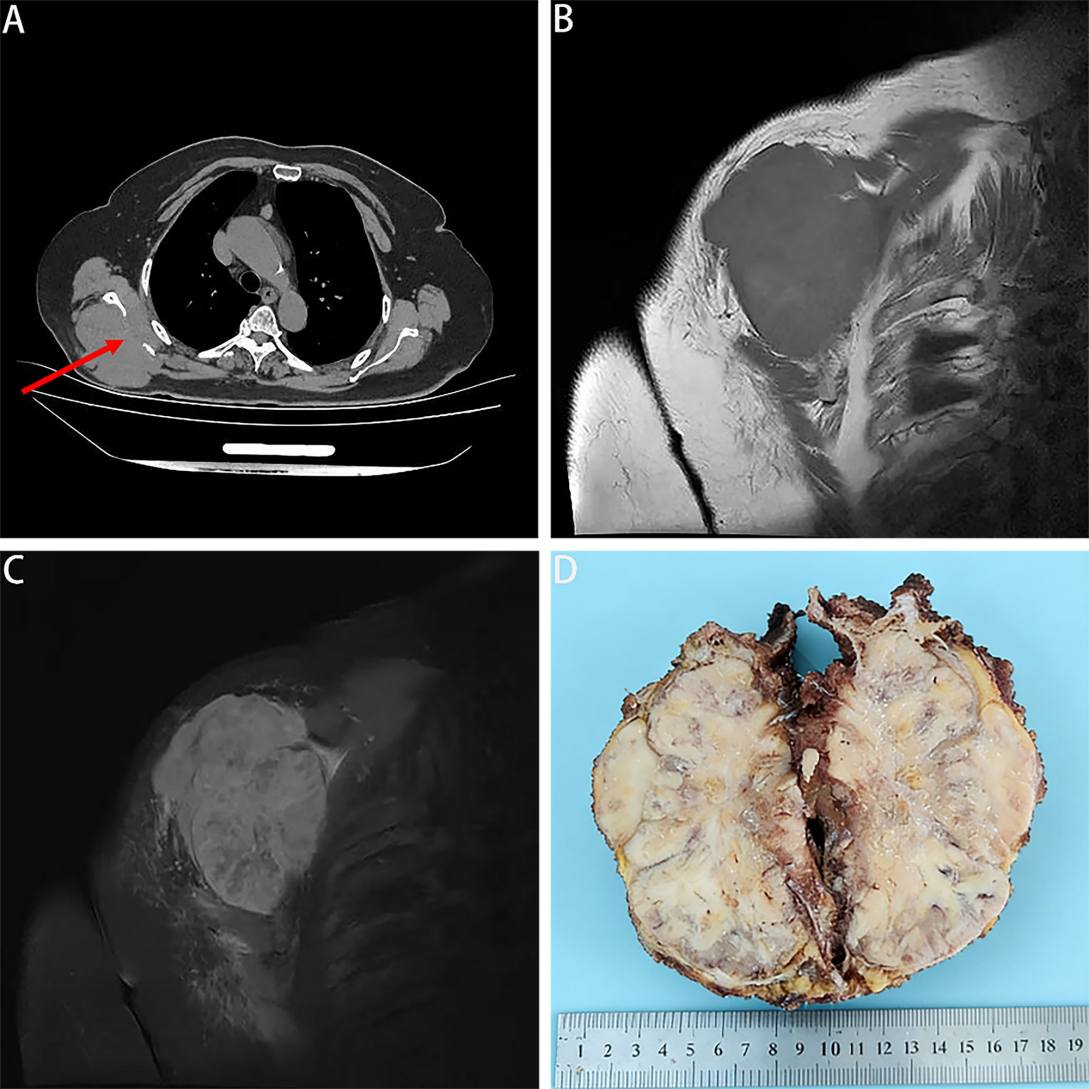
Radiologic and gross features of FRCT. **(A)** Computed tomography revealed an irregular soft tissue mass lateral to the right scapula, associated with destruction of the underlying bone; **(B, C)** Magnetic resonance imaging demonstrated an irregular mass lesion with ill-defined margins relative to the adjacent soft tissues. The lesion exhibited heterogeneous signal intensity, with mixed iso-/hypointensity on T1-weighted images **(B)** and mixed iso-/hyperintensity on T2-weighted images **(C)** signal intensity, involvement of the adjacent scapula was noted; **(D)** The cut surface of the tumor was grayish-yellow with irregular but well-demarcated borders.

A 67-year-old female with a 30-year history of severe hypertension underwent surgical resection for a mass on the posterior aspect of her right shoulder at another hospital in February 2016. Postoperative pathology revealed a low-grade malignant mesenchymal-derived tumor with a size of 7 cm × 6.6 cm × 5.5 cm. According to the 8th edition of the AJCC staging system for soft tissue sarcomas, the primary tumor was staged as T2N0M0 (clinical stage III A). In October 2023, a subcutaneous mass was noted again in the right shoulder and back. Physical examination revealed a subcutaneous mass measuring 12 cm × 8 cm, with firm texture, clear boundaries, poor mobility, mild tenderness, and slight limitation of shoulder joint movement. Computed tomography showed a right parascapular mass with heterogeneous attenuation and evidence of scapular destruction (**Figure 1A**). Magnetic resonance imaging demonstrated an irregular mass with ill-defined boundaries relative to the adjacent soft tissue. The lesion showed mixed isointense and hypointense signal on T1-weighted images and mixed isointense and hyperintense signal on T2-weighted images (**Figures 1B, C**).

**Figure 2:**
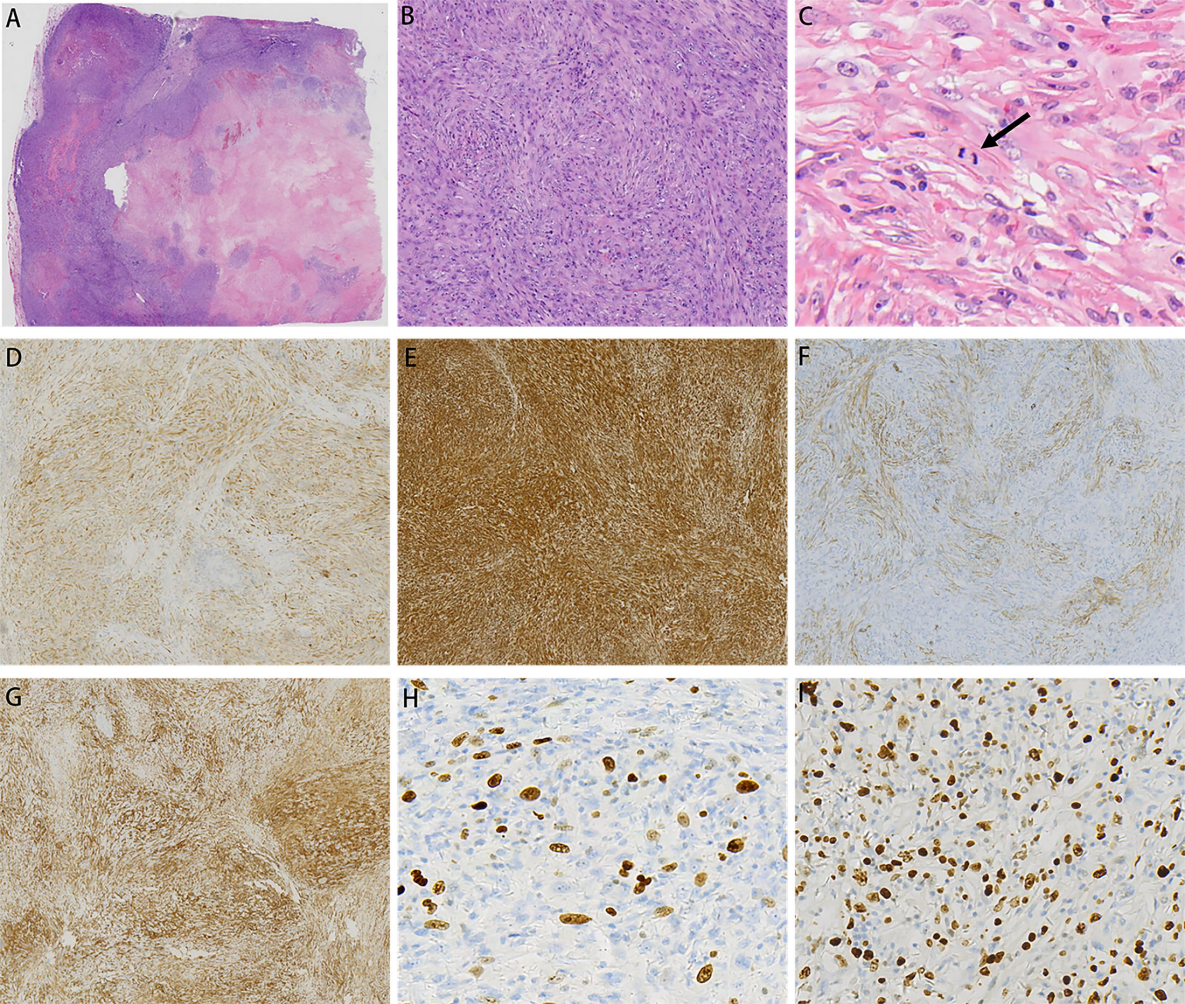
Histopathological and immunohistochemical features of FRCT. **(A)** The tumor was well−circumscribed and showed extensive geographic necrosis (Hematoxylin-Eosin (HE) staining, ×0.5); **(B)** The tumor comprised spindle cell arranged in a storiform pattern and sheets of epithelioid cells (HE staining, ×10); **(C)** Epithelioid cells with pale eosinophilic cytoplasm, moderate nuclear atypia, vesicular nuclei and prominent nucleoli (mitotic figures indicated by arrows) (HE staining, ×40); **(D–G)** Immunohistochemical staining showed the tumor cells were positive for CK **(D)**, vimentin **(E)**, and CD163 **(G)**, with focal positivity for SMA **(F)**; **(H, I)** The Ki−67 proliferation index was lower in the recurrent tumor [**(H)**, 25%] compared to the primary tumor [**(I)**, 35%].

Gross examination revealed a grayish-yellow mass measuring 12 cm × 9.8 cm × 7.6 cm within the resected bone and soft tissue specimen, with irregular but well-demarcated borders (**Figure 1D**). Histopathological examination showed a tumor composed of a mixture of diffuse proliferating spindle-shaped and epithelioid cells with moderate cytoplasm. The cells were arranged in storiform and sheet-like patterns. Tumor cell nuclei were ovoid with slightly coarse chromatin, distinct nuclear membranes and prominent nucleoli. Tumor giant cells and multinucleated cells were common. Mitotic count was approximately 3 per 10 high-power fields (HPFs), and extensive tumor necrosis was present (**Figures 2A–C**). A focal fibrous pseudocapsule was observed at the tumor periphery. The tumor cells showed diffuse positivity for cytokeratins (CK, CK8/18, CAM5.2), vimentin, CD68, CD163, and EMA, with focal expression of SMA, S-100, calponin, CD3 and CD43. They were negative for ALK, Desmin, CD34, CD21, CD23, CD35, CDlα, CD117, CD20, HHV-8 and p53 (**Figures 2D–G**; **Supplementary Figure 1**), which were similar to the immunohistochemical expression profile of the primary tumor. The Ki-67 labeling index was 25%, which was lower than that of the primary tumor (35%) (**Figures 2H, I**). *In-situ* hybridization for Epstein–Barr virus-encoded RNA was negative.

**Figure 3:**
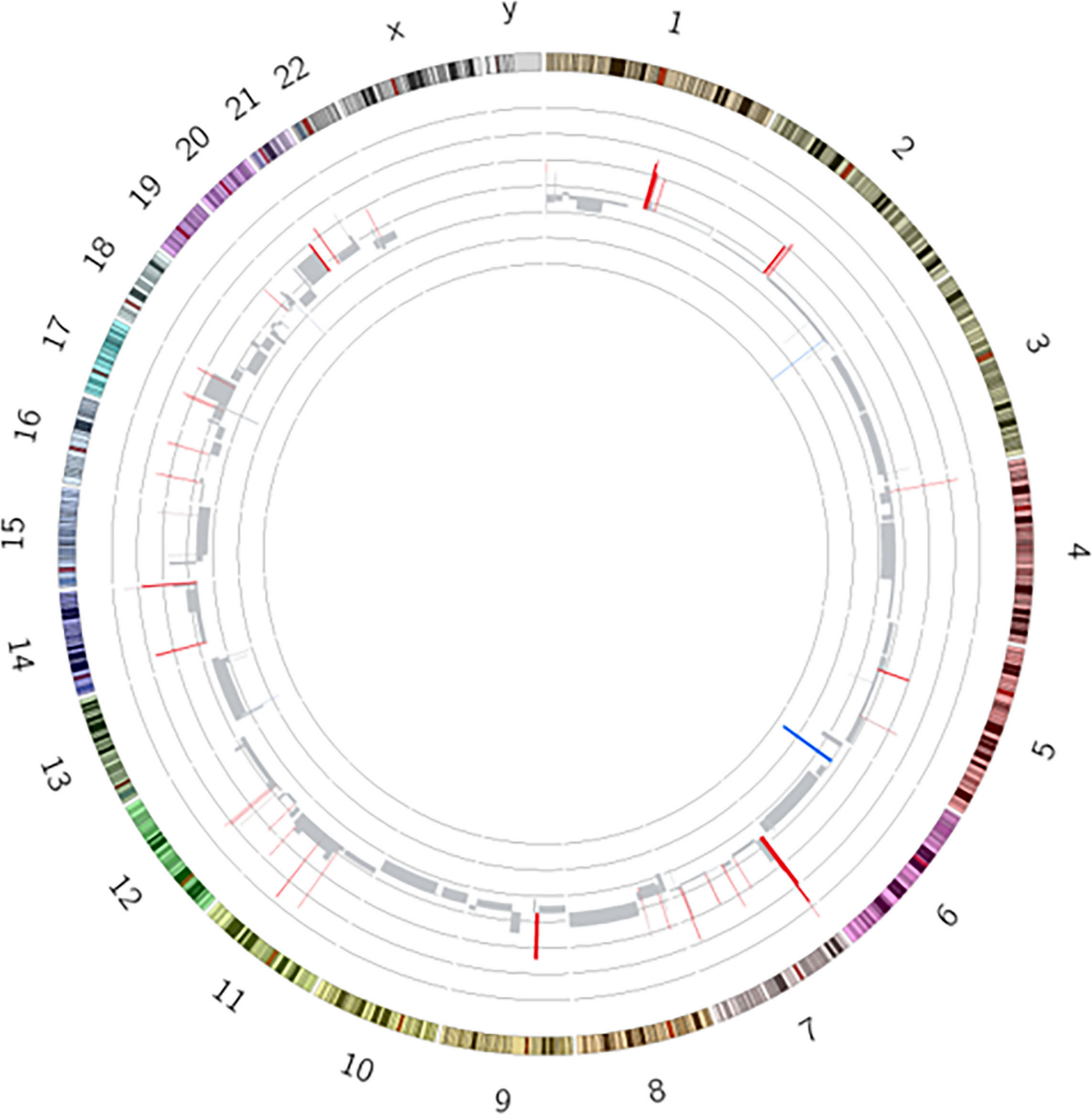
Copy number analysis by whole exome sequencing. Analysis revealed copy number gains on chromosomes 7, 11 and 14 in the tumor cells.

Ultimately, the diagnosis of FRCT/CIRC tumor was confirmed. As postoperative magnetic resonance imaging revealed no change in tumor stage (rT3N0M0, clinical stage III B, according to the 8th edition of the AJCC staging system for soft tissue sarcomas), the patient underwent observation. At the last follow-up in January 2026, the patient showed no evidence of disease progression and denied any additional symptoms or discomfort. WES and RNA sequencing were performed on the recurrent tumor sample and adjacent normal tissues. WES detected 30 single nucleotide variants and 7 small insertions and deletions in the tumor sample, with variant allele frequencies (VAF) ranging from 2.17% to 12.6% (**Table 1**). Gene copy number gains were observed on chromosomes 7, 11, and 14 (**Figure 3**). Microsatellite analysis indicated that the tumor was microsatellite stable. Detection of loss of heterozygosity in human leukocyte antigen (HLA) revealed two HLA heterozygous deletions: HLA-B *39:01:01:01 and HLA-C*07:02:01:01. The RNA sequencing used to detect gene breaks or fusions did not yield any results of gene fusion.

**Table 1:** The gene mutations and INDEL variant detected by whole exome sequencing in presentpt case.

Chr	Pos	Ref	Alt	Gene	cDNA	Protein	Variant classification	VAF
1	6589077	G	A	NOL9	c.1802C>T	p.Pro601Leu	Missense Mutation	0.053
1	16257527	AAAG	–	SPEN	c.4795_4798del	p.Glu1599LysfsTer32	Frame Shift Del	0.033
2	1895988	TGC	–	MYT1L	c.2102_2104del	p.Ser701del	In Frame Del	0.022
2	26534797	G	A	ADGRF3	c.1799C>T	p.Pro600Leu	Missense Mutation	0.110
2	112733051	T	A	MERTK	c.1144 + 2T>A		Splice Site	0.064
4	26417146	A	–	RBPJ	c.252del	p.Glu85AsnfsTer21	Frame Shift Del	0.027
5	140736169	G	A	PCDHGA4	c.1495G>A	p.Gly499Arg	Missense Mutation	0.045
5	148687042	C	T	AFAP1L1	c.613C>T	p.Arg205Ter	Nonsense Mutation	0.082
5	172195770	A	G	DUSP1	c.1099T>C	p.Cys367Arg	Missense Mutation	0.103
7	133812248	C	G	LRGUK	c.128C>G	p.Ser43Cys	Missense Mutation	0.024
8	6289098	–	A	MCPH1	c.321dup	p.Arg108ThrfsTer2	Frame Shift Ins	0.033
8	105463515	C	T	DPYS	c.382G>A	p.Asp128Asn	Missense Mutation	0.125
8	144461662	C	T	RHPN1	c.929C>T	p.Ala310Val	Missense Mutation	0.054
11	2337501	C	T	TSPAN32	c.586C>T	p.Gln196Ter	Nonsense Mutation	0.052
11	55944259	C	T	OR5J2	c.166C>T	p.His56Tyr	Missense Mutation	0.044
11	111414861	T	C	LAYN	c.323T>C	p.Leu108Pro	Missense Mutation	0.034
12	18747443	A	C	PIK3C2G	c.4027A>C	p.Ser1343Arg	Missense Mutation	0.066
12	51237640	C	T	TMPRSS12	c.203C>T	p.Pro68Leu	Missense Mutation	0.049
13	33344887	–	A	PDS5B	c.4169dup	p.Asn1390LysfsTer13	Frame Shift Ins	0.038
13	79219057	T	G	RNF219	c.148A>C	p.Ser50Arg	Missense Mutation	0.036
13	99457262	T	A	DOCK9	c.5821A>T	p.Ile1941Phe	Missense Mutation	0.065
14	74532023	A	G	ALDH6A1	c.1265T>C	p.Val422Ala	Missense Mutation	0.036
14	74532039	C	A	ALDH6A1	c.1249G>T	p.Glu417Ter	Nonsense Mutation	0.043
14	95030078	G	A	SERPINA4	c.259G>A	p.Ala87Thr	Missense Mutation	0.034
15	42694326	C	G	CAPN3	c.1529C>G	p.Pro510Arg	Missense Mutation	0.060
15	83434752	C	T	FSD2	c.1585G>A	p.Val529Met	Missense Mutation	0.042
16	30510701	G	A	ITGAL	c.2036G>A	p.Gly679Asp	Missense Mutation	0.041
17	36099482	G	A	HNF1B	c.493C>T	p.Arg165Cys	Missense Mutation	0.085
17	38927484	A	G	KRT26	c.446T>C	p.Ile149Thr	Missense Mutation	0.033
18	61584739	A	–	SERPINB10	c.226del	p.Arg76GlyfsTer7	Frame Shift Del	0.028
19	1434836	C	–	DAZAP1	c.1155del	p.Ala386ProfsTer43	Frame Shift Del	0.032
19	36113608	C	G	HAUS5	c.1757C>G	p.Pro586Arg	Missense Mutation	0.041
22	30032794	C	T	NF2	c.169C>T	p.Arg57Ter	Nonsense Mutation	0.072
22	50684352	G	C	HDAC10	c.1820C>G	p.Ala607Gly	Missense Mutation	0.126
X	34961442	G	A	FAM47B	c.494G>A	p.Arg165His	Missense Mutation	0.022
X	44969421	A	G	KDM6A	c.4259A>G	p.Asn1420Ser	Missense Mutation	0.031
X	136113479	C	T	GPR101	c.355G>A	p.Val119Ile	Missense Mutation	0.026

VAF, variant allele frequency.

## Discussion

Fibroblastic reticulum cells (FRCs) are critical stromal support cells within lymphoid organs. Their functions include forming cellular networks that guide immune cell migration and maintaining the structural integrity and microenvironmental homeostasis of lymph nodes (9, 14). These cells are highly responsive to microenvironmental stimuli; for instance, reactive hyperplasia of FRCs has been observed in tumor-draining lymph nodes and in HIV-related lymphadenopathy (15). Notably, comorbidities are common in FRCT. Among the 57 patients reported in the literature to date (including the present case), 10 patients presented with concurrent malignant tumors (e.g., breast, endometrial, or gallbladder carcinoma) (6–8, 16–22); in addition, two patients had autoimmune diseases (23, 24), three patients had congenital/genetic disorders (including Lynch syndrome) (25, 26), and two patients had hepatitis C virus infection (5, 27, 28). Based on these observations, we speculate that the FRCT pathogenesis might be influenced by a multifactorial synergistic effect. Persistent disruption of the immune microenvironment–triggered by underlying conditions such as malignancy or immune-mediated diseases–could contribute to the malignant transformation of FRCs from reactive hyperplasia. Nevertheless, the only comorbidity in the present patient was hypertension, and there is still no direct clinical evidence to validate this hypothesis. Furthermore, whether differences in individual immune status influence the biological behavior of FRCT remains to be investigated.FRCT exhibits prognostic heterogeneity. Although some cases of FRCT exhibit an indolent clinical course (11, 19, 29), the literature review revealed that 35.1% of the patients experienced tumor recurrence or metastasis, with a mortality rate of 22.8% and a median overall survival of only 13 months. This finding indicates that the malignant potential of this neoplasm has been underestimated to a certain degree. Accordingly, it may be more appropriate to view FRCT as a tumor spectrum, from indolent to highly aggressive. This understanding highlights the importance of combining pathological characteristics with molecular markers in clinical practice to inform individualized therapy and follow-up strategies.

The diagnosis and differential diagnosis of FRCT are highly challenging. This tumor is rare and poorly characterized from a pathological perspective. Some cases express cytokeratin with a lymphoplasmacytic background, which frequently leads to misdiagnosis. In the literature, 16 cases have been reported as initially misdiagnosed, most commonly as carcinoma or metastatic carcinoma (13, 19, 30–32), lymphoma (26, 33), or hemangioma (11, 34). Furthermore, FRCT exhibits marked morphological overlap with follicular dendritic cell sarcoma (FDCS) and interdigitating dendritic cell sarcoma (IDCS). All three tumors are composed of spindle and ovoid cells arranged in fascicular, whorled, or diffuse patterns, with necrosis and mitotic activity, and cannot be reliably distinguished by HE staining alone. Differential diagnosis therefore relies on morphology and immunohistochemistry. FRCT consistently expresses vimentin and is typically positive for α−SMA, desmin, CD68, and calponin; most cases express cytokeratin (e.g., pan−CK, CK8/18, CAM5.2). S100 is negative or shows focal weak positivity, in contrast to the diffuse strong S100 expression seen in IDCS. CD21 and CD35 are uniformly negative, effectively excluding FDCS. FDCS is diffusely positive for CD21/CD35 and negative for cytokeratin, α−SMA, and S100, and carries an intermediate prognosis. IDCS is diffusely positive for S100 and CD68, exhibits the most aggressive behavior and has the worst prognosis. FRCT displays striking prognostic heterogeneity: localized early-stage disease has a favorable outcome after resection, whereas advanced or metastatic disease carries a dismal prognosis, with a 2−year mortality rate of 100% and a median survival of only 13 months, which is significantly worse than that of FDCS and IDCS (13).The WES analysis of this case provided important experimental evidence for exploring the potential molecular drivers of FRCT pathogenesis and behavior. Previous literature has demonstrated that FRCT exhibits highly complex karyotypic abnormalities, such as 47, XXY and multiple complex clonal chromosomal aberrations, including hypertetraploid, hyperdiploid, and pseudodiploid karyotypes (9, 35). The present case exhibited copy number gains on chromosomes 7, 11, and 14, further supporting the notion that chromosomal instability may play a significant role in FRCT tumorigenesis and progression. Notably, amplification of these chromosomal regions may involve candidate genes associated with cell proliferation and immune modulation. Their potential correlations with genetic mutations and immune evasion merit further exploration in subsequent functional studies.

We further profiled the somatic mutational landscape of the tumor. The WES analysis of this case identified 30 somatic single-nucleotide variants and 7 small insertions/deletions, all with low VAFs (2.17%–12.6%). Similarly, somatic mutations in CCND1, CIC, FOXP1, and SOX17 (VAFs ranging from 2.26% to 9.34%) were identified in an adrenal FRCT (36). These low-VAF mutations may reflect tumor subclonal heterogeneity or random genetic alterations driven by chromosomal instability, although their exact biological functions and clinical implications remains to be elucidated.

Most notably, we identified two heterozygous deletions in the HLA genes (HLA-B*39:01 and HLA-C*07:02), a finding with significant biological and clinical implications. HLA LOH directly impairs the antigen presentation machinery, enabling tumor cells to evade host T-cell-mediated immune surveillance (37). A wealth of studies has demonstrated that immune evasion mediated by HLA LOH acts as a key driver for the onset and progression of multiple malignancies (38). Pan-cancer analyses have further verified that HLA LOH occurs in approximately 17% of cancer patients (39). Previous studies have shown that the prevalence of HLA LOH reaches up to 40% in lung cancer, which is closely linked to tumor metastasis and subclonal evolution. Of note, primary tumor regions with metastatic potential display a significantly lower effective neoantigen burden than non-metastatic regions in lung adenocarcinoma (37). Combined with the molecular characteristics of chromosomal instability and low-VAF mutations in this case, HLA LOH may serve as a critical driver event that endows tumor cells with immune evasion capacity against the background of genomic instability.

Correlating this molecular finding with the clinical course, we hypothesize that HLA LOH may represent a key mechanism underlying the tumor’s long-term latency and eventual recurrence following initial surgery. Furthermore, HLA LOH has potential guiding significance for FRCT treatment. Immune evasion resulting from HLA LOH may underlie the poor efficacy of immune checkpoint inhibitor monotherapy in FRCT (12, 17). Consequently, FRCT patients harboring HLA-LOH are unlikely to benefit from single-agent immune checkpoint inhibitor therapy. Therefore, alternative strategies to circumvent this immune evasion mechanism, such as combination regimens or novel cellular therapies, warrant investigation. Collectively, HLA LOH not only represents a key molecular feature driving immune evasion in this FRCT case but also holds promise as a predictive biomarker to inform individualized treatment decisions.

In conclusion, this study reports a recurrent case of FRCT, in which whole-exome sequencing detected a variety of low VAF genetic variations and copy numbers gains. We reported for the first time HLA LOH in this tumor. Chromosomal instability appears to be a prominent feature of FRCT. The identification of HLA LOH uncovers a potential immune evasion mechanism that may explain resistance to immune checkpoint inhibitors in this tumor. This alteration may serve as a biomarker to guide personalized treatment.

## Data Availability

The datasets presented in this study can be found in online repositories. The names of the repository/repositories and accession number(s) can be found below: OMIX [OMIX017193] (https://ngdc.cncb.ac.cn/omix/).

## Supplementary material

Supplementary Figure 1 Immunohistochemical features and differential diagnosis of FRCT. Immunohistochemically, tumor cells showed diffuse positivity for CAM5.2 **(A)**, CK8/18 **(B)**, EMA **(C)**, and CD68 **(D)**, with focal expression of Calponin **(E)**, CD3 **(H)**, CD43 **(I)**, and S-100 **(S)**. Tumor cells were negative for Desmin **(F)**, CD20 **(G)**, CD30 **(J)**, CD34 **(K)**, ALK **(M)**, CD117 **(N)**, HHV-8 **(O)**, CD21 **(P)**, CD35 **(Q)**, CD23 **(R)**, and CD1α **(T)**. *In situ* hybridization for Epstein–Barr virus-encoded RNA (EBER) was negative **(L)**.
