# Supplementary figures and images for: Case Report: Cytokeratin-positive interstitial reticulum cell tumor with HLA loss of heterozygosity
Source: Front Immunol. 2026 Jun 2;17:1844173. doi: 10.3389/fimmu.2026.1844173 (PMC13268894; doi:10.3389/fimmu.2026.1844173)

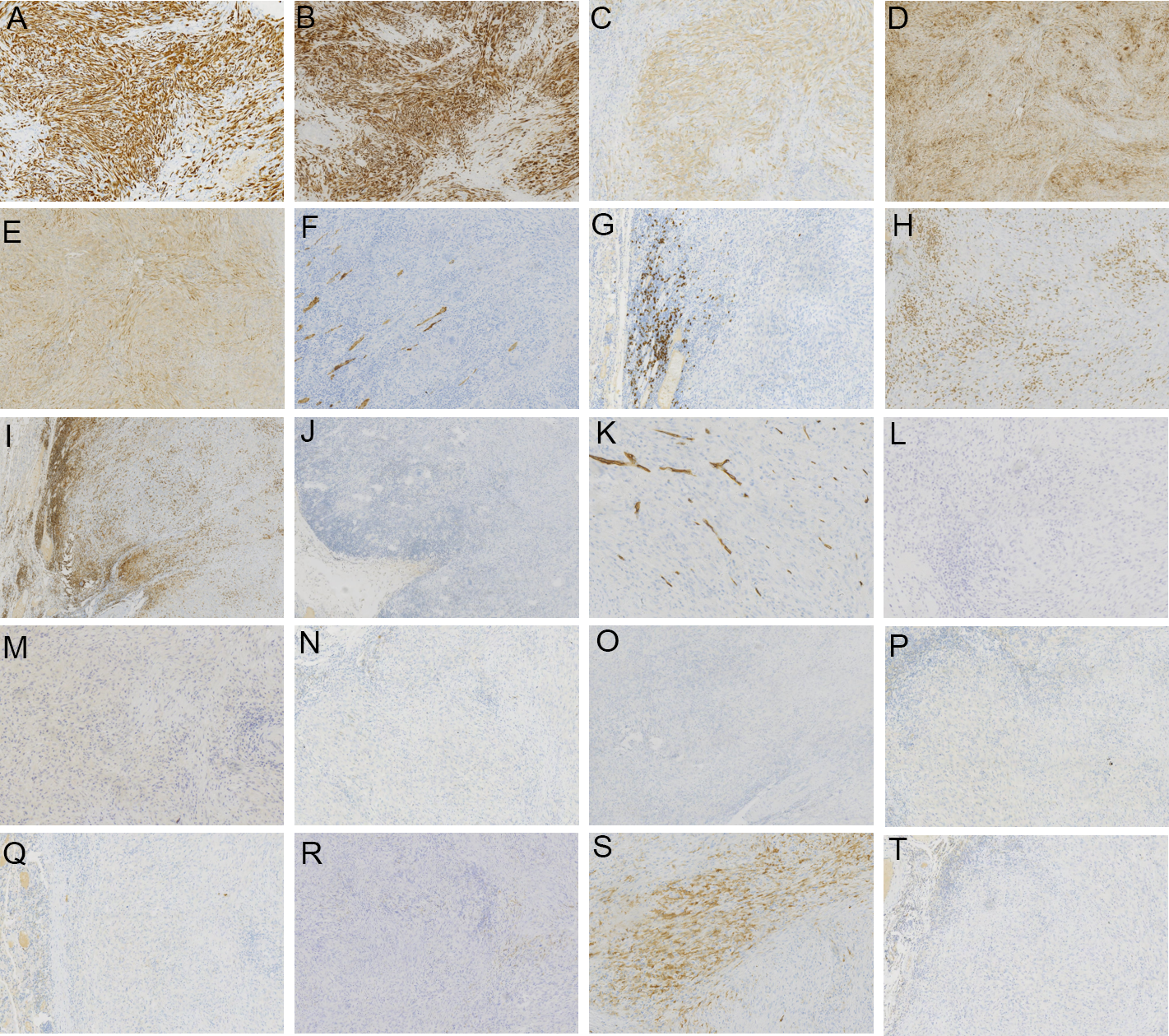

Supplement: Supplementary file 2 [file Image1.tif]
